# High dosage of agmatine alleviates pentylenetetrazole-induced chronic seizures in rats possibly by exerting an anticonvulsive effect

**DOI:** 10.3892/etm.2014.1711

**Published:** 2014-05-14

**Authors:** HUIQIN XU, FUYONG OU, PEI WANG, MANGDULA NAREN, DONGPEI TU, RONGYUAN ZHENG

**Affiliations:** 1 Department of Neurology, The First Affiliated Hospital and Research Institute of Experimental Neurobiology, Wenzhou Medical College, Wenzhou, Zhejiang 325000, P.R. China; 2 Department of Neurology, Chenzhou No. 1 People’s Hospital, Chenzhou, Hunan 423000, P.R. China

**Keywords:** agmatine, chronic seizures, pentylenetetrazole, N-methyl-D-aspartic acid receptor

## Abstract

The aim of the present study was to investigate the mechanism underlying the effects of different doses of agmatine in rats with chronic epilepsy. To generate chronic epilepsy models, rats pretreated with different doses of agmatine (20, 40 and 80 mg/kg) were intraperitoneally injected with pentylenetetrazole (35 mg/kg) for 28 consecutive days. Epileptic behavior was observed using behavioral measurements of convulsion for 1 h after each treatment with pentylenetetrazole. Morphological alterations of the hippocampal neurons were also observed using hematoxylin and eosin staining. In addition, the expression levels of glial fibrillary acidic protein and inducible nitric oxide synthase (iNOS) in the hippocampus were detected by immunohistochemistry. Furthermore, reverse transcription polymerase chain reaction was performed to detect the mRNA expression of two subunits (NR1 and NR2B) of the N-methyl-D-aspartic acid (NMDA) receptor in the rat hippocampus. The results demonstrated that administration of agmatine (80 mg/kg) significantly decreased the daily average grade of epileptic seizures and also reduced neuronal loss and astrocyte hyperplasia in the hippocampal area. Furthermore, agmatine (80 mg/kg) affected the mRNA expression levels of the NR1 subunit of the NMDA receptor, however, agmatine had no effect on the expression of iNOS in the hippocampus. Higher doses of agmatine inhibited the effect of pentylenetetrazole in rats, reduced astrocytic hyperplasia and neuronal damage in the hippocampus caused by seizures and selectively reduced the expression of the NR1 subunit of NMDA. Our results suggest that agmatine has an anticonvulsive effect in chronic epilepsy.

## Introduction

Agmatine is an endogenous amine synthesized via the decarboxylation of L-arginine mediated by arginine decarboxylase. Agmatine is expressed in a variety of animal organs, particularly the brain, where it acts as a novel neurotransmitter or neuromodulator (1).

Although the specific physiological actions of agmatine have yet to be elucidated, numerous studies have confirmed that agmatine significantly inhibits seizures induced by maximal electroshock and pentylenetetrazole in rat models (2–6). The selective reduction of N-methyl-D-aspartic acid (NMDA) receptor-mediated activity (3,4) and the inhibition of nitric oxide synthase activity (2,6) have been proposed to contribute to this inhibitory effect on seizures. However, the majority of previous studies have investigated the effect of agmatine on acute seizures, and only a few studies have used chronic epilepsy animal models, which are similar to the pathological physiology of clinical epileptic patients. It was hypothesized that agmatine may also have an anticonvulsive effect in chronic epilepsy. Therefore, in the present study, pentylenetetrazole-induced chronic epilepsy rat models were employed in order to examine the anticonvulsive effects of agmatine.

## Materials and methods

### Animals

A total of 50 healthy, male adult Sprague-Dawley rats (weighing between 170 and 200 g; The Experimental Animal Center of Wenzhou Medical College, Wenzhou, China) were used in the present study. Convulsion was induced in the rats using pentylenetetrazole (Sigma, St. Louis, MO, USA) as previously described (7). Pentylenetetrazole (35 mg/kg) was administered to rats in the agmatine pretreatment and model control groups each morning via intraperitoneal injection for 28 consecutive days. The present study was approved by the ethics committee at the Medical University of Wenzhou (Wenzhou, Zhejiang, China) and was in accordance with the Chinese laws for animal protection.

The rats were randomly divided into the following five groups, with 10 rats in each group: i) the saline-saline group (normal control group), saline was injected as the negative control; ii) the pentylenetetrazole-saline group (model control group), the rats were treated with saline 30 min prior to intraperitoneal injections of pentylenetetrazole; iii) the pentylenetetrazole low-dose agmatine group (pentylenetetrazole + 20 mg/kg agmatine; Sigma); iv) the pentylenetetrazole medium-dose agmatine group (pentylenetetrazole + 40 mg/kg agmatine); and v) the pentylenetetrazole high-dose agmatine group (pentylenetetrazole + 80 mg/kg agmatine). Agmatine pretreatment was administered 30 min prior to the pentylenetetrazole injections.

### Behavioral observations of convulsion

The behavior of each rat was observed for 1 h after pentylenetetrazole injection. The seizure activity was scored according to the following five-point scale as previously described by Fathollahi *et al* (8): stage 0, no response; stage 1, ear and facial twitching; stage 2, convulsive wave throughout the body; stage 3, myoclonic jerks; stage 4, turn onto their side; stage 5, turn over onto their back, generalized tonic-clonic seizures. The convulsion grade of each rat was recorded daily. If the rat maintained the epileptic behavior (i.e. at stage 2) for five consecutive days, it was regarded as kindling and the kindling rate was calculated.

### Sample preparation

After the rats were decapitated, the entire brain was rapidly removed and dissected on ice. One half of the hippocampus was immediately flash frozen in liquid nitrogen and stored at −80°C for subsequent reverse transcription polymerase chain reaction (RT-PCR) experiments. The other half was immersed in 4% paraformaldehyde (Shanghai Generay Biotech Co., Ltd., Shanghai, China) for 24 h at 4°C and then paraffin embedded. The paraffin-embedded brain was then cut into 5-μm thick coronal sections using a microtome. For each rat, several brain sections were collected for subsequent experiments.

### Hematoxylin and eosin staining

Two paraffin slices were selected and stained using hematoxylin and eosin as previously described (9). In the slices, hippocampal CA3, CA1 and DG regions were examined using a light microscope (BX51M; Olympus, Tokyo, Japan; magnification, ×10) to observe morphological alterations of the hippocampal neurons.

### Immunohistochemistry

Immunostaining was performed on the brain slices using the Polink-2 Plus^®^ HRP Polymer Detection System (PV-9001; GBI Labs, Mukilteo, WA, USA) as previously described (9). Then, the sections were briefly dehydrated through a graded series of ethanol and incubated with rabbit anti-mouse glial fibrillary acidic protein (GFAP) antibody (Santa Cruz Biotechnology, Inc., Santa Cruz, CA, USA). The sections were washed with phosphate-buffered saline (Shanghai Generay Biotech Co., Ltd.) and then incubated with poly horseradish peroxidase anti-rabbit secondary antibody (PV-9001). The avidin-biotin complex and diaminobenzidine were used to obtain a visible reaction product. As a negative control, the specimens in the control experiments were processed without primary or secondary antibodies. The immunostaining of inducible nitric oxide synthase (iNOS) was performed in a similar manner, however, the primary antibodies were substituted with rabbit anti-mouse iNOS antibody (Santa Cruz Biotechnology, Inc.). A Leica microscope equipped with a digital camera was used for the examination and imaging of the sections (Leica, Solms, Germany).

### Image analysis

To quantify the GFAP expression, the average number of positive cells in each section were counted in a blinded manner in five randomly selected high power fields in the hippocampal CA1 and CA3 areas (magnification, ×20), and plotted using Prism 3.0 software (GraphPad Software, Inc., San Diego, CA, USA). To quantify the iNOS expression, Image-Pro Plus 6.0 (Media Cybernetics, Inc., Rockville, MD, USA) was used to analyze the iNOS immunohistochemical images and to calculate the average light density values (IOD/area) of each section of five randomly selected high power fields in the hippocampal CA1 and CA3 areas (magnification, ×40). All the sections were analyzed under the same light intensity and magnification.

### RT-PCR

RT-PCR was performed using the Quant One Step RT-PCR kit (Tiangen Biotech, Co., Ltd., Beijing, China) according to the manufacturer’s instructions. The thermal cycler parameters were: 4 min at 94°C followed by 30 cycles of 30 sec at 94°C, 30 sec at 58°C, 40 sec at 72°C and then 10 min at 72°C. The following specific primers were used: NR1, forward 5′-GCTGCACGCCTTTATCTG-3′ and reverse 5′-TCCTACGGGCATCCTTGT-3′; NR2b, forward 5′-CACGGTGCCTTCAGAGTT-3′ and reverse 5′-CCTCCTCCAAGGTGACAA-3′. The PCR products were separated using electrophoresis on a 2.0% agarose gel. The intensity of the bands was analyzed using BioSense SC-810 Gel Documentation System (Shanghai Bio-Tech Co., Ltd., Shanghai, China) and Gel-Pro 3.1 software (Media Cybernetics, Inc., Bethesda, MD, USA).

### Statistical analysis

Statistical analysis was performed using SPSS 180 statistical software (SPSS, Inc., Chicago, IL, USA). The values are expressed as the mean ± standard error of the mean. Comparisons among multiple groups were performed using a one-way analysis of variance and a least significant difference post hoc test. P<0.05 was considered to indicate a statistically significant difference.

## Results

### Agmatine treatment reduces the severity of pentylenetetrazole-induced chronic seizures

**Figure 1:**
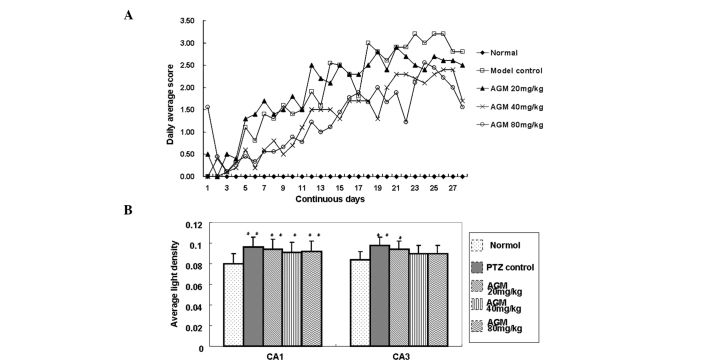
(A) Daily behavior scores for each group. The daily average scores of the 40 and 80 mg/kg agmatine groups were significantly decreased compared with the model control group (P=0.025 and 0.02, respectively). (B) Average light density (IOD/Area) values of inducible nitric oxide synthase expression in the CA1 and CA3 regions of the hippocampus (CA1, P<0.05; CA3, P<0.01; compared with the normal control group). ^*^P<0.05, ^**^P<0.01. AGM, agmatine; PTZ, pentylenetetrazole.

To evaluate the effect of agmatine on chronic seizures induced by pentylenetetrazole, convulsions were measured using a five-point scale, as previously described by Fathollahi *et al* (8). Following 20 days of treatment, the majority of the rats reached a completely kindled condition. The daily average seizure grades in the 40 and 80 mg/kg agmatine groups were significantly lower compared with those of the model control group (P<0.001; Fig. 1A). However, no significant difference in the kindling rate was observed among the agmatine and model control groups (Table I).

### Agmatine has no effect on the expression of iNOS

To investigate the effect of agmatine on the expression of iNOS, the average light density (IOD/Area) values of iNOS-positive regional expression in the CA1 and CA3 areas of the hippocampus were obtained. The data revealed that the pentylenetetrazole group had significantly higher values compared with the normal group (P<0.05). A decreasing trend was observed in the agmatine-treated rats compared with the rats in the model control group, however, no significant difference was observed. This suggested that iNOS activity may be increased in chronic epileptic seizures, however, the long-term usage of agmatine does not significantly inhibit iNOS expression (Fig. 1B).

### Agmatine treatment decreases cell injury in the hippocampal area of pentylenetetrazole-treated rats

**Figure 2:**
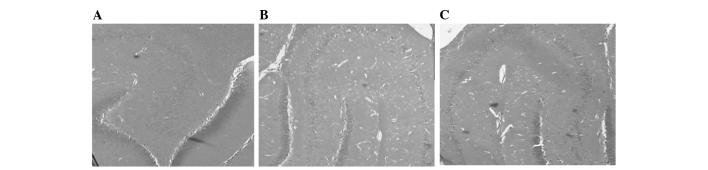
Decreased neuronal injury in the hippocampal region of agmatine-treated rats. The hippocampus region of rats was sectioned and stained using hematoxylin and eosin to compare the severity of neuron loss between the (A) normal control, (B) model control and (C) the agmatine groups (magnification, ×4).

To determine whether agmatine alleviated cell injury in the hippocampal area of pentylenetetrazole-treated rats, hippocampal pyramidal cells were observed under a microscope. In the normal group, hippocampal pyramidal cells exhibited regular morphological integrity, whereas, in the model control group, cell loss was observed and the cells were irregularly distributed and exhibited abnormal structures, as well as wider interspaces. By contrast, the agmatine group also exhibited neuronal loss, however, with reduced severity, particularly in the hippocampal area (Fig. 2). This observation indicated that agmatine treatment partially decreased cell injury in the hippocampal area.

### Treatment with agmatine suppresses astrocytic hyperplasia

**Figure 3:**
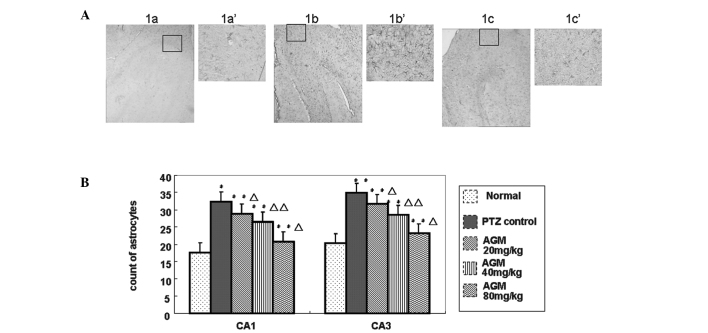
(A) Hyperplasia of astrocytes in the hippocampus of agmatine-treated rats was reduced. Immunostaining of GFAP was performed on the sections to detect increased astrocyte expression. The expression of astrocytes in the (1a and 1a′) normal control group and the (1c and 1c′) agmatine group was decreased compared with the (1b and 1b′) model control group (magnification, ×4; boxed area, magnification, ×20). (B) The number of GFAP positive cells was counted from five randomly selected microscopic fields (magnification, ×20) and plotted. Data are presented as the mean ± the standard deviation. ^*^P<0.05, ^**^P<0.01, compared with the normal control group. ^Δ^P<0.05, ^ΔΔ^P<0.01, for comparisons between the agmatine group and the model control group. AGM, agmatine; PTZ, pentylenetetrazole; GFAP, glial fibrillary acidic protein.

To investigate how agmatine treatment affects astrocytic hyperplasia, hematoxylin and eosin staining and image analysis were performed (Fig. 3A). In the CA1 and CA3 hippocampal regions of normal rats, GFAP-positive cells were scattered, light brown-yellow in color and reduced in number. By contrast, in the model control group, GFAP-positive cells were significantly increased in number and exhibited more intense staining, as well as thicker and extended neurites. In the agmatine groups, GFAP expression was increased to a certain extent, however, the number of GFAP-positive cells was reduced and the staining was less intense compared with the model control group. The cells were decreased in size and the neurites were relatively thinner and shorter. The differences between the agmatine groups and the model control group were significant (P<0.05), in particular for the 40 and 80 mg/kg agmatine groups (P<0.01), as shown in Fig. 3B. These results demonstrated that agmatine suppressed astrocytic hyperplasia.

### Agmatine treatment decreases the expression of the NMDA receptor

**Figure 4:**
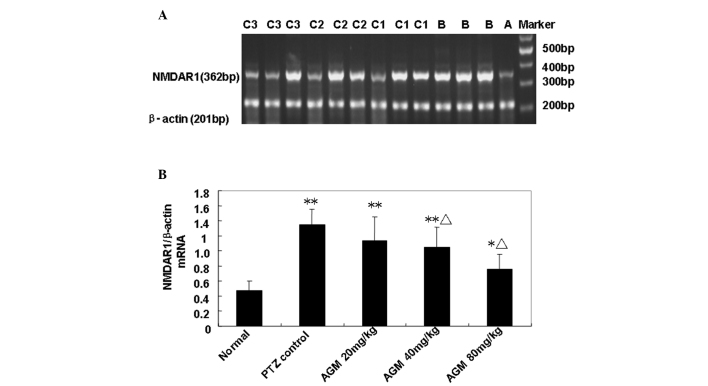
(A) Detection of NMDAR1 mRNA expression in the rat hippocampus using reverse transcription polymerase chain reaction. A, normal control group; B, model control group; C1, C2 and C3, agmatine groups (20, 40 and 80 mg/kg, respectively). (B) Quantification of NMDAR1 mRNA expression of the five groups. The Y axis indicates the ratio of optical density (OD) of the samples to the corresponding internal standard (β-actin). Data are expressed as the mean ± standard error of the mean (n=10). ^*^P<0.05, ^**^P<0.01, compared with the normal control group. ^Δ^P<0.01, for comparisons between the agmatine group and the model control group. NMDAR1, N-methyl-D-aspartic acid receptor; AGM, agmatine; PTZ, pentylenetetrazole.

In order to analyze the alterations in the expression of the NMDA receptor induced by agmatine, RT-PCR was performed to detect NR1 and NR2b mRNA expression in the rat hippocampus (Fig. 4A). Compared with the model control group, the quantity of NR1 mRNA in the agmatine groups (40 and 80 mg/kg) was significantly decreased (P<0.01), suggesting that pretreatment with agmatine may suppress the actions of the hippocampal NR1 (Fig. 4B). However, the low-dose agmatine group (20 mg/kg) showed no significant difference compared with the model control group (Fig. 4B). In addition, no significant difference in NR2b mRNA expression was observed among all the groups (data not shown). These results indicated that treatment with higher concentrations of agmatine decreased the expression of the NMDA receptor.

## Discussion

In the present study, it was demonstrated that consecutive administration of agmatine provided protection against pentylenetetrazole-induced chronic seizures in rats. These results are consistent with previous studies that demonstrated the inhibitory effect of agmatine in acute seizure animal models (2,3). Furthermore, in the present study, rats treated with agmatine exhibited significantly reduced astrocytic hyperplasia and neurological defects in the hippocampal area compared with rats in the model control group. Furthermore, the expression of the NMDA1 receptor was selectively suppressed in agmatine-treated rats.

The results from the present study are in accordance with several previous studies demonstrating that high doses of agmatine had marked anticonvulsive effects (3–5). In the present study, only rats treated with a high dose of agmatine (40 and 80 mg/kg/d) demonstrated clear inhibitory effects. This may be due to the rapid metabolism of agmatine in the peripheral tissues. In addition, the blood-brain barrier may also restrict the penetration of agmatine into the brain (6). Therefore, an adequate peripheral dose is required to produce apparent protective effects. However, this is controversial as certain studies have found that agmatine administered alone at doses of ≤100 mg/kg had no affect on the threshold and provided no protection against seizures (7). In fact, in the present study repeated administration of agmatine did not decrease the kindling rate. This suggests that agmatine is unable to alter the threshold and this may be associated with under-dosing, which requires investigation in future studies.

Astrocytes are important glial cells in the brain. Following epilepsy, the number of astrocytes increases and alterations in morphology and function are observed. Astrocytes have been demonstrated to be important in the mechanisms underlying epilepsy (10). For example, it has been demonstrated that they are involved in the maintenance of the inflammatory state during epilepsy by releasing inflammatory cytokines. These cytokines directly alter the excitability of the neurons and promote mossy fiber budding of the dentate gyrus to form an excitability loop, which may induce seizures (11). In the present study, GFAP immunohistochemistry demonstrated that agmatine was able to significantly reduce hippocampal astrocytic cell proliferation in a dose-dependent manner following pentylenetetrazole-induced seizures. This may contribute to the inhibitory effect of agmatine on seizures.

The activation of NMDA receptors is responsible for the development of seizures and their binding sites are upregulated in different types of convulsant animal models. NMDA receptor antagonists have previously been demonstrated to inhibit convulsion (12). In addition, agmatine has been shown to selectively modulate NMDA subunits in rat hippocampal neurons (12) and mediate anticonvulsive actions (3,4). In accordance with previous studies, RT-PCR results from the present study demonstrated that the mRNA expression of NR1 was significantly inhibited in the agmatine groups (40 and 80 mg/kg). However, this was only observed in animals repeatedly treated with large doses of agmatine. The reason for this may be that only 1% of the injected agmatine reaches the brain (4). However, the same result was not observed for NMDA R2b mRNA expression.

Seizures are known to cause neuronal death and cell loss may in turn increase the potential for further seizure activity. This feedback cycle may explain the progressive and chronic course of epilepsy (13). Previous studies have revealed a decrease in the number of hippocampal neurons in seizures induced by pentylenetetrazole (14). In the present study, the results of hippocampal morphology suggest that agmatine may decrease cell loss in the rat hippocampus. Agmatine exhibits seizure-suppressive and neuroprotective capabilities and may therefore protect against convulsions on seizure-suppressive and neuroprotective capabilities.

Several studies have also suggested that the inhibitory effect of agmatine may be important in its anticonvulsive properties (2,6). In the present study, the expression of iNOS was found to increase in the hippocampus following pentylenetetrazole administration. However, agmatine was not found to significantly inhibit iNOS expression.

In conclusion, the present study demonstrated that agmatine has a protective effect against pentylenetetrazole-induced chronic seizures and that its effective dose is relatively large (80 mg/kg). Agmatine treatment results in decreased astrocytic hyperplasia, neuronal cell loss and selective suppression of the NMDA1 receptor in the hippocampus. The majority of clinical epilepsy cases are diagnosed as long-term repeated chronic epilepsy. Thus, further investigation regarding the function of agmatine in chronic epilepsy is particularly important.

## Figures and Tables

**Table I:** Severity and the kindling rate of rats in each treatment group.

			Seizure grade		
				
Group	Sample (n)	Survival rate	Moderate (≤III)	Severe (≥III)	Kindling rate
Normal control (A)	10	-	-	-	-
Model control (B)	10	10/10	5/10	5/10	10/10
Agmatine 20 mg/kg (C1)	10	10/10	6/10	4/10	10/10
Agmatine 40 mg/kg (C2)	10	10/10	6/10	4/10	10/10
Agmatine 80 mg/kg (C3)	10	10/10	7/10	3/10	8/10

The rate of severe grade seizures in agmatine groups was markedly decreased compared with the model control group, whilst no significant difference was observed between the kindling rates among the groups.
